# Prevalence and patterns of third molar impaction among Ethiopians in Addis Ababa: a retrospective pilot study

**DOI:** 10.1038/s41598-024-59821-x

**Published:** 2024-04-18

**Authors:** Tsedenia Gebeyehu, Yeshewas Abaynew

**Affiliations:** 1 Atlas College of Health Sciences, Addis Ababa, Ethiopia; 2 School of Public Health, College of Medicine and Health Sciences, Wollo University, Dessie, Ethiopia

**Keywords:** Impacted, Pattern, Maxilla, Mandible, Molar, Diseases, Health care, Medical research

## Abstract

Tooth impaction is a condition in which a tooth does not reach its normal position and is often observed in the third mandibular molar due to inadequate space. This study aimed to investigate the prevalence and configuration of the impacted third molars with an emphasis on angular orientations in a sample of the Ethiopian population. This cross-sectional study included a retrospective analysis of 291 patient records and orthopantomography data from the archives of a private dental clinic in Addis Ababa, during the study period from December 2020 to November 2022. Demographic details and data on the position and level of the impacted third molars were evaluated using the Winter classification. Data were analyzed for frequency distribution. The prevalence of impacted third molars was 22% (n = 64), with a greater incidence on the right side (60.9%) and a higher frequency in the mandible (67.2%). Vertical angulation (32.8%), followed by mesioangular angulation (31.2%), was the most common impaction pattern. The results highlight the need for improved treatment protocols for third molar impaction, emphasizing the prevalence in the mandible and the importance of addressing vertical impaction. Regular dental check-ups are essential for assessing third molar impaction and planning appropriate management. These data can inform policymaking and treatment considerations for impacted third molars in the Ethiopian population.

## Introduction

Tooth impaction, a pathological condition characterized by the failure of a tooth to reach its normal functional position, is notably more prevalent in the third molar than in other teeth. Specifically, among humans, the third molar mandibular molars exhibit the highest frequency of impaction. The appearance of impacted third molars is a routine discovery in dental practice, and effective dental treatments are effective in addressing this condition^1^.

Several factors contribute to the higher impaction rate of the third molars, including insufficient space, limited skeletal growth, an enlarged crown size, and delayed maturation of these molars^2^. Among all teeth, the third mandibular molars are the most commonly impacted^3^. In addition, impacted teeth are more frequently encountered in the maxilla than in the mandible^3^.

Impaction of the third mandibular molar frequently arises from a lack of space between the distal aspect of the second mandibular molar and the anterior border of the ascending ramus of the mandible. Impacted third molars are associated with various complications, including pericoronitis, pain, caries, bone loss, incisor crowding, resorption of adjacent tooth roots, trismus, food impaction, and cheek biting^1,2,4–6^.

Impaction of the third molar manifests itself as different angulations. According to Winter’s classification, third molars can be impacted in a vertical, mesioangular, horizontal, or distoangular orientation^7,8^.

The prevalence of impacted third molars varies significantly worldwide. For example, a study in Eritrea^9^ reported a prevalence of mandibular impaction of 15.2%, while in Hong Kong^10^, it was significantly higher, at 27.8%. In Japan^7^, the reported prevalence was 24.3%; in Korea^11^, it was 53.9%; in Yemen^12^, it was 38.8%; in Iran^13^, it was 23%; and in Saudi Arabia^7^, it was reported as 24.3%.

A study carried out in southeastern Iran^14^ revealed that the most common angulation of impaction in the mandible was mesioangular impaction (48.3%), followed by horizontal impaction (29.3%), while the most common angulation of impaction in the maxilla was vertical impaction (45.3%), followed by mesioangular impaction (22.2%).

In Saudi Arabia^15^, vertical angulation was most common in the maxilla (56.5%), followed by distoangular angulation (31.9%), and in the mandible, mesioangular angulation (40.5%) was most common, followed by vertical angulation (32.0%).

In Hong Kong^10^, more than 80% of the third impacted mandibular molars were horizontally mesioangularly angulated, and in Japan^11^, vertical angulation was found primarily in the maxillary teeth (50%), while mesioangular angulation was found primarily in the mandible (48.3%).

Several previous studies have revealed that men and women have a significant difference in the magnitude of third molar impaction^14,16,17^. An Iraqi study^18^ established a close correlation between the impaction of the third molars and the sex and age of the patients. On the contrary, other studies^19,20^ have not reported significant differences in impaction status between men and women.

This study aimed to investigate the prevalence and configurations of impacted third molars, including an examination of their angular alignment. Understanding the prevalence and configurations of impacted third molars in a given region is an important clinical issue, as impacted teeth are predisposed to periodontal diseases, and it is helpful to treat this problem early to reduce associated complications.

Limited data exist on the prevalence and patterns of impacted third molars in the Ethiopian population. This study aims to address this gap by determining the prevalence and patterns of impacted third molars, specifically at a private Dental Clinic in Addis Ababa. The findings of this research aim to establish baseline data, providing valuable information that can inform the development of improved strategies for the management of impacted third molars.

## Results

### Sociodemographic characteristics of the participants

A total of 291 patient records were reviewed; 178 (61.2%) were women, while 134 (46%) of the study participants were 15–29 years old (Table 1).

**Table 1:** Sociodemographic characteristics of study participants in Addis Ababa, 2022/23.

Variables	Third molar impaction		Percent (%)
	Yes	No	
Sex			
Male	33	80	38.2
Female	31	147	61.2
Age groups			
15–29	38	96	46
30–44	14	107	41.6
> = 45	12	24	12.4

### Prevalence of third molar impaction

**Figure 1:**
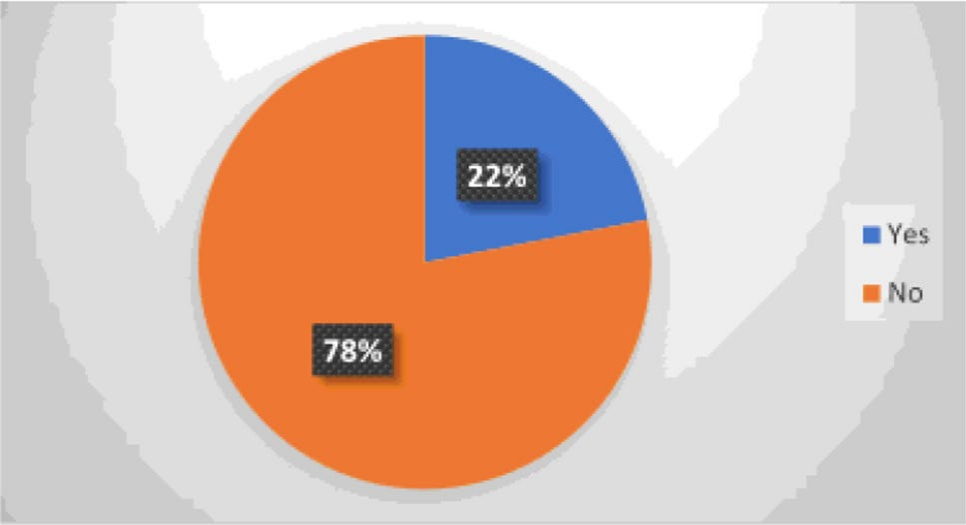
Prevalence of impacted third molars among patients in Addis Ababa, 2022/2023.

Among the 291 patients, 64 (22%) had at least one impacted third molar (Fig. 1). The 64 patients with third molar impaction were in all three age groups.

### Pattern of third molar impaction

Among the 64 patients with impacted third molars, vertical impaction was the most prevalent at 32.8%, followed by mesioangular impaction at 31.2% (Table 2).

**Table 2:** Angulation of the impacted third molars in Addis Ababa, 2022/23.

Angulation of impacted third molars	Frequency	Percent (%)
Mesioangular	20	31.2
Vertical	21	32.8
Horizontal	12	18.8
Distoangular	11	17.2

In terms of the appearance of third molar impactions of the maxillary and mandibular, impaction of the third molar of the mandible took precedence, accounting for 67.2% of cases compared to maxillary impaction (Table 3).

**Table 3:** The distribution of impacted third molars between the maxilla and the mandible in Addis Ababa, 2022/23.

Site of impaction	Frequency	Percentage (%)
Mandible	43	67.2
Maxilla	15	23.4
Both	6	9.4

There was a significantly higher incidence of third molar impaction on the right side (60.9%) than on the left side (Table 4).

**Table 4:** Location of third molar impaction among patients in Addis Ababa, 2022/23.

Variable	Frequency	Percentage (%)
Location of impaction		
Right	39	60.9
Left	16	25.0
Both	9	14.1

## Discussion

This retrospective review of the records aimed to assess the frequency and distribution of the impacted third molars among patients who sought dental care at a private dental clinic in Addis Ababa.

Third molar impaction can create significant challenges during surgery, including limited visibility and access, and an increased risk of nerve damage. Impacted molars can also cause problems, such as infection, pain, cysts, and tumors, affecting overall oral function. Regular dental checks and timely evaluation of affected molars are crucial in preventing or managing these potential problems.

This study revealed that the prevalence of affected third molars was 22%, which is consistent with the findings of a previous study conducted by Yildirim and Büyükgöze-Dindar in 2022 in Turkey^21^, where the prevalence was reported to be 23%. However, the prevalence observed in this study was lower than that reported in several other studies. For example, in a study conducted by Alfadil and Almajed Saudi Arabia in 2020^15^, 58.3% of the participants had impacted third molars, while in a study conducted by Jain et al. in 2029 in India^22^, 52.3% of orthodontic patients had at least one impacted third molar. Similarly, a study by Al-Shamahy in 2019 in Yemen^12^ and a study by Al-Anqudi et al. in 2014 in Oman^23^ reported prevalence rates of 38.8% and 54.3%, respectively, for individuals with at least one affected third molar. These variations may be attributed to differences in the study populations across the various studies.

In this study, the highest prevalence of third molar impaction was found at the age of 15 to 29 years, which could be because most third molars erupt at this age. In this study, the prevalence of third molar impaction was highest in females. Similarly, a study conducted by Ishwarkumar et al. in 2019 in South Africa^5^ reported a higher frequency of third molar impaction in female patients. This indicates that third molar impaction is more common in women, which is related to factors such as jaw size, hormonal changes, and genetic influences that can contribute to the observed differences between men and women.

This study revealed a higher prevalence of vertical impaction (32.8%), followed closely by mesioangular impaction (31.2%). A similar trend was observed in a study conducted by Ayranci et al. in 2017 in Turkey^24^, where the vertical angulation was reported to be 57.3%, significantly surpassing that of other angulations. Similarly, a study conducted by Anjum et al. in 2014 in Pakistan indicates vertical (26%) and mesioangular (59%) impacts are the most prevalent^8^.

However, contrasting findings were observed in some studies, a study conducted by Arefi et al. in 2022^25^ reveals that the most common impaction pattern was mesioangular in the mandible and distoangular in the maxilla. In a study conducted by Passi et al. in 2019^26^ among the Delhi Delhi-National Capital Region population, mesial angulation emerged as the most prevalent presentation in impacted mandibular third molars at 49.2%, with a vertical position observed in 24% of cases. This variation could be related to age, genetic, and clinical variations of studies.

Furthermore, a study conducted by Hassan AH in 2010 in India^19^ reported that the predominant mandibular impaction is mesioangular at 60%. Previous studies conducted by Padhye et al. in 2013 in the Indian population^27^ and Ramamurthy et al. in 2012 in South India^28^ also highlighted mesioangular impaction as the most common pattern. The inconsistent frequency of angulation among these studies may be attributed to variations in age, ethnic and racial groups, genetics, and clinical characteristics between the investigated populations.

This study revealed a greater incidence of third molar impaction in the mandible (67.2%) than in the maxilla. This finding is consistent with previous studies, including a retrospective study by Hashemipour et al. in 2013 in southeast Iran^14^, which reported a significantly higher percentage of impacted third molars in the mandible (54.9%) than in the maxilla (28.8%). Similarly, a study conducted by Alfadil and Almajed in 2020 in Saudi Arabia^15^ reveals that impaction was more common in the mandible (58.5%) than in the maxilla (41.5%). A study conducted by Chu et al. in 2003 in Hong Kong^10^ also revealed that impaction of the mandibular third molars was the most prevalent (82.5%), followed by that of the maxillary third molars (15.6%). Furthermore, a study conducted by Ayranci et al. in 2017 in Turkey^24^ reported a significantly higher proportion of impacted mandibular third molars (57.3%) than of impacted maxillary third molars (42.7%). The probability of impacted mandibular third molars was 1.33 times higher than that of impacted maxillary third molars. These rate variations could be attributed to factors such as different ethnic and age groups studied, eruption times, sample sizes, pathological conditions, or variations in radiographic criteria for dental development and eruption.

This study revealed that the right side (60.9%) was more affected than the left side. This finding is consistent with a retrospective study conducted by Alfadil et al. in 2020 in Saudi Arabia^15^ that reported a statistically significant difference between prevalence on the right (59.9%) and left side (40.1%). Furthermore, another study conducted by Anjum et al. in 2014 in Pakistan^8^ showed that impaction was more common on the right side of the mandible. Furthermore, a study conducted by Raj Kumar et al. in 2017 in Eritrea^9^ showed that the right side had a higher prevalence than the left. However, in a study conducted by Chu et al. in 2003 in Hong Kong^10^, the results revealed that the distribution of the impacted teeth was similar between the left and right sides. This difference could be attributed to variations in the racial and ethnic groups of the subjects who participated in the research, as well as genetic code and sociodemographic characteristics. The findings of this study will provide evidence that clinicians should make the appropriate diagnoses and implement better treatment protocols to avoid further complications.

### Strengths and limitations of the study

It is great that the study used a pre-tested instrument administered by trained data collectors, which contributed to the enhanced validity of the results. However, the limitations you have pointed out are crucial considerations for the interpretation of the study findings.

1. Limited generalizability: The focus of the study on patients attending private dental clinics may limit the generalizability of the findings to the broader population. Individuals who did not visit dental clinics regularly, particularly those of lower socioeconomic status, were not included in the study. This could introduce selection bias, affecting the external validity of the results.
2. Single-clinic setting: Conducting the study in a single private dental clinic may not provide a complete picture of the characteristics of third molar impaction. Dental clinic populations can vary, and results from a single clinic may not reflect the diversity present in different settings.
3. Exclusion of individuals: Mentioning that a substantial number of people were not included in the study raises questions about the representativeness of the sample. Understanding the reasons for exclusion and the potential impact on the results is essential for evaluating the study's internal validity.
4. Retrospective study design: The retrospective nature of the study design introduces inherent limitations, such as reliance on historical data and potential issues related to the completeness and accuracy of the data.

Readers and researchers should be aware of these constraints when considering the applicability of the findings to different populations or settings. While the study benefited from certain strengths, the limitations mentioned underscore the need for caution when generalizing the results. Future research could address these limitations by including a more diverse sample involving multiple clinics and employing a prospective study design.

## Conclusions

The results of this study should be interpreted with caution in light of these limitations. The following conclusions were drawn:

1. Twenty-two percent of the patients had an affected third molar.
2. There was a lower prevalence of impacted third molars in the maxilla than in the mandible.
3. Vertical impaction was more prevalent than other impaction patterns.
4. Large-scale community-based studies conducted in diverse geographic, environmental, and socioeconomic settings are recommended to determine the prevalence and patterns of impacted third molars.

## Materials and methods

### Study area and period

The research was conducted at Dr. Emebet Special Higher Dental Clinic, located in Yeka Subcity, Addis Ababa, and founded in 1991 E.C. Recognized as one of Ethiopia's premier private dental facilities, it boasts a team of skilled professionals, including ten dentists, one dental therapist, two orthodontists, two implantologists, one radiologist, five laboratory technicians, and 24 nurses. Data were collected from December 1 to 30 December 2022.

### Study design

A retrospective cross-sectional study was conducted at a private dental clinic in Addis Ababa to examine the prevalence, patterns, and factors associated with impacted third molars among patients. The investigation focused on gathering information from historical data within the institutional setting.

### Population

The source population for this study included all dental patients receiving treatment at a private dental clinic. On the other hand, the study population comprised patients who had been treated in the clinic from December 2020 to November 2022. Individuals with inadequate-quality orthopantomography, who experienced trauma, or who showed pathology in the jaw that could impact the alignment of the dentition were excluded from the study. Furthermore, patients whose third molars had no root filling were not included in the study.

### Determination of the sample size

The sample size for this study was calculated using the population proportion formula, considering a confidence level of 95%, a margin of error of 5%, a prevalence of third molar impaction of 24.4% based on previous research^29^, a source population of 6000, and factoring in a nonresponse rate of 10%. As a result, the sample size determined for the study was 291 patients.

### Sampling method

Study participants were selected using a systematic random sampling technique, employing a sampling interval of 20. To start the process, a random start was determined through simple random sampling, and the starting point was identified as the fourth participant. Subsequently, every 20th study participant in the sequence was included in the study. This method helped ensure a representative and unbiased sample from the larger population.

### Data collection procedure

The data collection instrument for this study was developed after a meticulous review of the available literature. It was originally drafted in English and was subjected to a pre-test phase to ensure its effectiveness before being implemented in the main study. The data collection process entailed a thorough examination of patient records. Trained data collectors, supervised by designated personnel, were tasked with summarizing the information gleaned from these records. To assess the position and depth of the impacted teeth, the principal investigator conducted a panoramic radiograph analysis in collaboration with experts. The Winter classification system served as the framework to assess the location and depth of the impacted teeth.

### Data entry and analysis

The data entered were processed using Statistical Package for the Social Sciences (SPSS version 20, IBM Corp., USA) and are presented in text, frequency tables, and graphs.

### Data quality assurance

To ensure data quality, a pre-tested collection instrument was employed, which was administered by trained collectors and supervised by personnel. Experts interpreted the panoramic radiograph results. Daily reviews ensured completeness and consistency, with necessary data editing performed to exclude missing information.

### Ethical approval

Ethical approval for the study was obtained from the Atlas College Health Sciences Research Ethics Committee. A letter of cooperation was extended to Dr. Emebet’s Special Higher Dental Clinic. The study team secured a grant to carry out the research. Additionally, the institution gave informed consent, allowing access to patients' medical records.

### Ethics approval and consent to participate

The study received ethical approval from the Atlas College of Health Sciences Research Ethics Committee. The ethics committee also granted informed consent from participants, which was waived by the committee that approved the study. All methods were carried out in accordance with the Declaration of Helsinki.

## Data Availability

The dataset utilized in this article is incorporated within the article itself.
